# From Left Atrial Dimension to Curved M-Mode Speckle-Tracking Images: Role of Echocardiography in Evaluating Patients with Atrial Fibrillation

**DOI:** 10.31083/j.rcm2305171

**Published:** 2022-05-11

**Authors:** Hao-Tien Liu, Hui-Ling Lee, Chung-Chuan Chou

**Affiliations:** ^1^Division of Cardiology, Department of Internal Medicine, Chang Gung Memorial Hospital, Linkou branch, 33304 Taoyuan, Taiwan; ^2^Department of Anesthesia, Chang Gung Memorial Hospital, Taipei branch, 10507 Taipei, Taiwan; ^3^School of Medicine, Chang Gung University College of Medicine, 33302 Taoyuan, Taiwan

**Keywords:** atrial fibrillation, catheter ablation, echocardiography, left atrial enlargement, left atrial emptying fraction, deep learning neural networks

## Abstract

Left atrial (LA) enlargement and dysfunction increase the risk of atrial fibrillation (AF). Traditional echocardiographic evaluation of the left atrium has been limited to dimensional and semi-quantification measurement of the atrial component of ventricular filling, with routine measurement of LA function not yet implemented. However, functional parameters, such as LA emptying fraction (LAEF), may be more sensitive markers for detecting AF-related changes than LA enlargement. Speckle-tracking echocardiography has proven to be a feasible and reproducible technology for the direct evaluation of LA function. The clinical application, advantages, and limitations of LA strain and strain rate need to be fully understood. Furthermore, the prognostic value and utility of this technique in making therapeutic decisions for patients with AF need further elucidation. Deep learning neural networks have been successfully adapted to specific tasks in echocardiographic image analysis, and fully automated measurements based on artificial intelligence could facilitate the clinical diagnostic use of LA speckle-tracking images for classification of AF ablation outcome. This review describes the fundamental concepts and a brief overview of the prognostic utility of LA size, LAEF, LA strain and strain rate analyses, and the clinical implications of the use of these measures.

## 1. Introduction

Atrial fibrillation (AF) is the most prevalent symptomatic cardiac arrhythmia in clinical practice worldwide. AF increases the risk of ischemic stroke, heart failure, cardiovascular events, and mortality [1, 2, 3, 4]. Atrial fibrosis has been increasing recognized as a contributing abnormality in the development of AF [5, 6, 7]. Atrial fibrosis increases local conduction heterogeneity in the atria and provides a AF-sustaining re-entry substrate [7, 8], which can be identified by delay-enhancement cardiac MRI and intracardiac electroanatomic mapping [9, 10]. However, their time-consuming and invasive nature limit the routine application of these tools in daily practice. Echocardiography provides a real-time and noninvasive method to assess cardiac anatomy and function. Because of its widespread availability and feasibility, echocardiography has been the imaging technique of choice for evaluating the left atrium. Several echocardiographic parameters of left atrial (LA) anatomy, function, and deformation have been used to evaluate atrial fibrosis and the risk of AF [1, 11, 12].

Catheter ablation (CA) is a common treatment strategy in symptomatic AF patients resistant to antiarrhythmic medications, but the long-term success rate is only around 50–80% [13, 14, 15]. LA remodeling is among the most important factors related to the recurrence of AF post CA. Previous studies have investigated the clinical predictors of AF recurrence after CA [16, 17, 18]. P-wave duration can serve as a low cost and widely available predictor of long-term outcome in AF patients undergoing CA [19, 20, 21, 22]; nevertheless, the predictive power of P-wave duration is weaker than that of LA emptying fraction (LAEF) [21]. Echocardiography has the advantages of availability, efficacy, and providing real-time high temporal and spatial resolution images, and thus is best suited for evaluating the possibility of AF recurrence [23, 24, 25, 26].

In this review, we provide a comprehensive overview of the LA echocardiographic parameters associated with new-onset AF and AF recurrence after CA.

## 2. Review of Parameters

### 2.1 Left Atrial (LA) Size Assessment

Left atrial dimension (LAD) in M-mode measurement is the traditional method used to assess LA size. LA dilatation reflects the cumulative effects of left ventricular (LV) filling pressure over time and the severity of diastolic dysfunction, and can be used as a quantifiable surrogate of the arrhythmogenic substrate in the development of AF. Previous studies have shown that dilated LAD is a predictor of AF occurrence in general [27] and in elderly populations [1], and that the risk of developing AF is proportionate to the extent of LA dilatation [28]. The Cardiovascular Health Study revealed that patients with LAD >50 mm had a four-fold higher risk of new-onset AF during surveillance [29]. For this unidimensional measurement of LAD to accurately represent the true LA size, it must be assumed to have a consistent relation with other LA dimensions [30]. However, the left atrium is not a spherical cavity and LA enlargement may occur asymmetrically [31], which results in underestimation of the LA size when using the anterior-posterior diameter acquired from M-mode images [30]. The American Society of Echocardiography and the European Association of Cardiovascular Imaging recommend using a biplane method to measure LA volume (LAV), using either the area-length technique or Simpson’s method [32]. Biplane LAV has been reported to predict AF occurrence in elderly population [1], in patients with cardiomyopathy [33], and in those with stroke of undetermined source [34]. Tsang* et al*. [35] also found that LAV is more powerful than LAD in predicting AF occurrence in the elderly population.

### 2.2 LA Function Assessment, Atrial Myopathy, and Atrial Fibrillation (AF) Genesis 

In normal subjects, LA function can be divided into three phases: reservoir, conduit, and booster pump, which account for around 40%, 35%, and 25% of the entire LV filling, respectively [11]. To assess LA function, LA volumes are measured at the mitral valve opening (${\mathrm{LAV}}_{\text{max}}$), closure (${\mathrm{LAV}}_{\text{min}}$), and at the onset of the electrocardiographic P wave (${\mathrm{LAV}}_{\text{preA}}$, only available in sinus rhythm); the LA functions are derived from the following volumetric measurements [36, 37]:

(1) $$\mathrm{LAEF}=\left({\mathrm{LAV}}_{\max}-{\mathrm{LAV}}_{\min}\right)/{\mathrm{LAV}}_{\max}$$

(2) $$\mathrm{LA}\,\text{ conduit function }=\left({\mathrm{LAV}}_{\max}-{\mathrm{LAV}}_{\text{preA }}\right)/{\mathrm{LAV}}_{\max}$$

(3) $$\text{ LA booster pump function }=\left({\mathrm{LAV}}_{\text{preA }}-{\mathrm{LAV}}_{\min}\right)/{\mathrm{LAV}}_{\text{preA }}$$

Clinically LAEF is a significant echocardiographic parameter for predicting AF occurrence. Cauwenberghs *et al*. [38] demonstrated that LAEF is a significant predictor of cardiac events and of new-onset AF. The area under the curve (AUC) of the receiver operating characteristic curve was 0.80 (95% confidence interval [CI] 0.73–0.88) for new-onset AF at 8 years of follow-up. A 55.5% cutoff value of LAEF had a sensitivity of 0.77 and specificity of 0.72 for predicting new-onset AF. The Copenhagen City Study also reported that not only enlarged ${\mathrm{LAV}}_{\text{max}}$ and ${\mathrm{LAV}}_{\text{min}}$ but also impaired LAEF were associated with an increased risk of AF in the general population; in individuals without hypertension, only LAEF was an independent predictor in all regression models; indeed, LAEF could even predict AF in individuals with a structurally normal left atrium (${\mathrm{LAV}}_{\text{max}}$ <34 mL/$m^{2}$) [39]. Abhayaratna *et al*. [40] reported that LAEF ≤49% was associated with risk for first AF independent of ${\mathrm{LAV}}_{\text{max}}$, LV function, and clinical factors in elder persons after a mean follow-up period of 1.9 ± 1.2 years. A subsequent analysis in the same cohort revealed that ${\mathrm{LAV}}_{\text{min}}$ may be a slightly more robust predictor of the development of AF [41]. Because a reduced LAEF is determined by an increased ${\mathrm{LAV}}_{\text{min}}$ for any given ${\mathrm{LAV}}_{\text{max}}$, ${\mathrm{LAV}}_{\text{min}}$ could be a better predictor of AF occurrence than ${\mathrm{LAV}}_{\text{max}}$.

Several reports have highlighted the role of atrial fibrosis in AF pathogenesis [7, 8, 42]. The development of fibrosis results in atrial myopathy [6], which is associated with atrial dysfunction and conduction disturbance [43]. Sung *et al*. [44] reported that ${\mathrm{LAV}}_{\text{max}}$ and ${\mathrm{LAV}}_{\text{min}}$ were significantly correlated with the percentage of low voltage area (LVA) in the left atrium and that LAEF was inversely correlated with the percentage of LVA. LA dysfunction caused by the effects of inflammation, oxidative stress, and atrial fibrosis plays an important role in AF development and progression [45, 46]. Once AF develops, rapid atrial depolarization leads to changes in ion channel function and electrical conduction, which shorten the atrial refractory period and further promote AF. A substudy of the ENGAGE-TIMI 48 trial evaluated LA size and function according to the electrical burden of AF as well as the stroke risk and reported that increasing abnormalities in LA structure and function were associated with a greater AF burden and greater risk of stroke [47]. Seewoster *et al*. [48] also reported that patients with persistent AF had larger LAV and worse LAEF than those with paroxysmal AF (PAF).

### 2.3 LA Anatomical and Functional Parameters in Predicting AF Ablation Outcomes

LA size may be a predictor of AF recurrence after CA. A meta-analysis of 22 studies revealed that dilated LAD increases the risk of AF recurrence after CA regardless of the follow-up duration [49]. Moreover, McCready *et al*. [23] demonstrated that a LAD cutoff value of 43 mm predicted long-term success following CA for those with persistent AF, with a sensitivity of 92% and a specificity of 52%. Similar results have been reported for LAV. In the meta-analysis by Njoku *et al*. [25] large LAV and LAV index (LAVI) increased the odds (odds ratio [OR] 1.032, 95% CI 1.012–1.052) and were independent predictors of AF recurrence post CA. Shin *et al*. [50] reported that LAV was the only predictor of AF recurrence after CA in multivariate analysis, and a LAVI cutoff value of 34 mL/$m^{2}$ showed a sensitivity of 70% and a specificity of 91% to predict AF recurrence. Kohari* et al*. [51] studied 125 patients with non-PAF undergoing pulmonary vein antral isolation and revealed that ${\mathrm{LAV}}_{\text{min}}$ index of 26 mL/$m^{2}$ and ${\mathrm{LAV}}_{\text{max}}$ index of 42 mL/$m^{2}$ were the best single parameters of AF recurrence after CA; but only ${\mathrm{LAV}}_{\text{min}}$ index and AF duration were the independent parameters for AF recurrence in multivariate analysis.

Several studies have demonstrated that LAEF is useful in predicting the maintenance of sinus rhythm in patients with AF post CA [52, 53, 54]. Our group demonstrated that LAEF, but not LAD or LAV, provides optimal prognostic information for risk stratification in 483 AF patients undergoing CA, which implies that LA dysfunction is an earlier indicator of atrial remodeling than LA dilatation [55]. Oka *et al*. [56] demonstrated the superiority of pre-ablation baseline LAEF over LAVI in predicting AF recurrence after CA in 292 patients with PAF undergoing single or multiple procedures. Charitakis *et al*. [54] investigated the association of the risk of AF recurrence with echocardiographic parameters (${\mathrm{LAV}}_{\text{max}}$ and LAEF), markers of cardiac endocrine function, as well as proteins related to inflammation, fibrosis, and apoptosis in 189 patients undergoing CA for AF. They found that patients with high concentrations of MR-proANP, CASP8, and NT3, and low LAEF (instead of ${\mathrm{LAV}}_{\text{max}}$) were at higher risk for recurrence, which implies that the LAEF and inflammation, fibrosis, and apoptosis related protein levels are better markers of AF-related changes than ${\mathrm{LAV}}_{\text{max}}$.

### 2.4 Left Ventricular (LV) Diastolic Function 

The LV diastolic phase could be divided into early rapid filling, diastasis, and atrial systole. There is a close interaction between the left atrium and LV diastolic function. Increased LV filling pressure reduces passive emptying volume from the left atrium to the left ventricle, triggering a compensatory mechanism that increases the active emptying volume by enhancing active LA contraction in the late diastole period [28]. Therefore, structural and functional LA remodeling is often the consequence of LV diastolic dysfunction [57]. Several echocardiographic parameters have been suggested as useful in evaluating LV diastolic function, such as LAVI, transmitral E/A ratio, isovolumic relaxation time, decelerating time of mitral early velocity, e’ on tissue Doppler imaging, and E/e’ and tricuspid regurgitation velocity [57, 58]. Although both ${\mathrm{LAV}}_{\text{max}}$ and ${\mathrm{LAV}}_{\text{min}}$ gradually increase with the progression of LV diastolic dysfunction, LAVmin may be a more sensitive marker of LV diastolic dysfunction than LAVmax [59]. Furthermore, recent studies have shown that LA strain changes progressively with the severity of LV diastolic dysfunction, and this parameter could reflect LA changes earlier than LAVI in patients with LV diastolic dysfunction [58, 60].

LV diastolic dysfunction adversely affects LA structural, functional, and electrical remodeling [61]; therefore, patients with the diagnosis of LV diastolic dysfunction have an increased risk of AF [62]. Tsang *et al*. [63] demonstrated that the risk of incident AF was proportionate to the severity of LV diastolic dysfunction, and LAVI was the strongest predictor of AF in the 840 elderly patients studied. Rosenberg *et al*. [64] used data from the Cardiovascular Health Study to analyze the influence of echocardiographic diastolic parameters on the risk of AF. They found that early mitral inflow velocity (peak E velocity), late mitral inflow (A-wave) velocity-time integral, and LAD were the predictors of incident AF. Vasan* et al*. [65] examined the diastolic parameters in patients in the longitudinally followed Framingham Heart Study and found that an E/e’ ratio greater than the median (1.23) increased the rate of incident AF. Arai *et al*. [66] revealed that an E/e’ ≥11.0 was associated with new-onset AF when adjusted for the coexistence of atherothrombotic risk factors, but the association was attenuated after adjustment for LAD.

Heart failure with preserved ejection fraction (HFpEF) is characterized by elevated LV filling pressures with clinical signs and symptoms of heart failure, LV diastolic dysfunction and a LV ejection fraction ≥50% [67]. HFpEF is associated with AF because of sharing similar risk factors and close link to diastolic dysfunction. Santhanakrishnan *et al*. [68] examined that temporal association of AF with HFpEF and heart failure with reduced ejection fraction (HFrEF) in the Framingham Heart Study participants with new-onset AF or heart failure. They found that AF was more likely to antedate rather than to follow heart failure, and prevalent AF preceded HFpEF in a higher proportion than HFrEF, possibly due to the similar pathophysiology that causes AF and HFpEF and reduced tolerance of individuals predisposed to HFpEF to AF during exertion to trigger clinical recognition of heart failure [69]. The persistence of elevated LV filling pressure causes LA remodeling and dysfunction [70], but LA remodeling may differ between HFpEF and HFrEF. By combining invasive pressure and noninvasive echocardiographic studies, Melenovsky* et al*. [71] revealed that patients with HFrEF had larger LAV and more depressed LA contractile function than HFpEF; but patients with HFpEF were characterized by larger LA pressure pulsatility, higher LA stiffness, and greater LA wall stress variation, which may contribute to a higher percentage of AF in the HFpEF group than in the HFrEF group (42% vs. 26%, *p* = 0.02). Note that using echocardiography to diagnose HFpEF in the setting of AF is challenging because of overlapping changes in echocardiographic parameters. For example, a dilated and impaired left atrium in sinus rhythm as a cardinal feature to reach the diagnosis of HFpEF may be pre-existing in PAF [72].

Parameters of LV diastolic dysfunction may serve as surrogate markers for AF recurrence post CA. Cha* et al*. [73] demonstrated an increased relative risk of AF recurrence of 1.8 (95% CI 1.1–3.1) in systolic dysfunction and 1.7 (95% CI 1.0–2.7) in isolated diastolic dysfunction compared with normal function at 1 year after CA. Kumar *et al*. [74] in a study of 124 patients undergoing CA for AF, found that high-grade LV diastolic dysfunction, defined by e’ on tissue doppler imaging and deceleration time, was an independent predictor of AF recurrence after adjustment for AF type and LAV (Hazard Ratio [HR] 2.6, *p* = 0.009). However, Kosiuk *et al*. [75] demonstrated that the E/A ratio and decelerating time could predict AF recurrence during the first week after CA, but that long-term results were not influenced by pre-procedural echocardiographic parameters that indicate LV diastolic dysfunction. A possible explanation for this finding is that LV diastolic function may deteriorate after AF ablation, mediated by longer ablation time, with a subsequent impact on LA and LV hemodynamics [76]. In addition, Nedios *et al*. [31] revealed that LA asymmetry was associated with LA dilatation and LV diastolic dysfunction and correlated with reduced success after AF ablation, but the presence or the grade of LV diastolic dysfunction was not associated with procedural success. Further investigations are needed into the definition and precise cutoff values to identify LV diastolic dysfunction and into the influence of LV diastolic dysfunction on AF recurrence after CA.

### 2.5 Total Atrial Conduction Time Measured by Tissue Doppler Imaging

Total atrial conduction time (TACT) is an atrial conduction parameter affected by atrial conduction velocity and anatomy [77]. The gold standard method of TACT measurement is intracardiac measurement using invasive electrophysiologic study [78]. Alternatively, TACT can be estimated noninvasively by PA-TDI interval, which is defined as the time interval between the onset of P wave on the surface electrocardiogram and the peak of A’ wave on tissue Doppler imaging [79]. Erdem *at al*. [78] revealed that TACT measured by PA-TDI correlated with that measured via invasive electrophysiologic study. Prolonged PA-TDI interval reflecting atrial remodeling [80, 81] has been shown to increase the risk of AF in various cohorts [82, 83, 84, 85]. Vos *et al*. [86] reported that a prolonged PA-TDI interval is vulnerable to new-onset AF in patients with various cardiovascular diseases with a HR of 1.375 per 10 ms increase in PA-TDI interval. Muller *et al*. [84] revealed that patients with prolonged PA-TDI intervals in the cryptogenic stroke cohort had higher incidences of AF detection. The AUC of the receiver operating characteristic curve was 0.94 for occult AF detection, and a PA-TDI interval cutoff value of 145 ms had a sensitivity of 93.8% and a specificity of 90.5% for identifying occult AF at 1-year follow-up. Leung *et al*. [81] investigated the relation between echocardiographic markers of LA fibrosis and AF progression in patients with new-onset AF (620 subjects) and controls (342 subjects). They found that PA-TDI interval and LA reservoir strain were correlated negatively, and patients with persistent AF had a longer PA-TDI interval and smaller LA reservoir strain than those with PAF. In predicting AF recurrence after successful electrical cardioversion or CA, Mueller *et al*. [87] demonstrated that PA-TDI interval at a cutoff value of 152 ms had a sensitivity of 87% and a specificity of 100% for predicting early AF recurrence after successful cardioversion in patients with non-PAF; Uijl* et al*. [88] demonstrated that PA-TDI interval had a better discriminative performance than ${\mathrm{LAV}}_{\text{max}}$ index (AUC 0.765 vs. 0.561, respectively) in predicting AF recurrence after CA. Karantoumanis *et al*. [89] also revealed that measurement of PA-TDI interval at different walls of the left atrium provides good performance (AUC ranging from 0.975–0.994) with a sensitivity of 98% and a specificity of 100% at a mean PA-TDI interval cutoff value of 125.8 ms for predicting AF recurrence after CA.

### 2.6 Speckle-Tracking Echocardiography

**Fig. 1.**
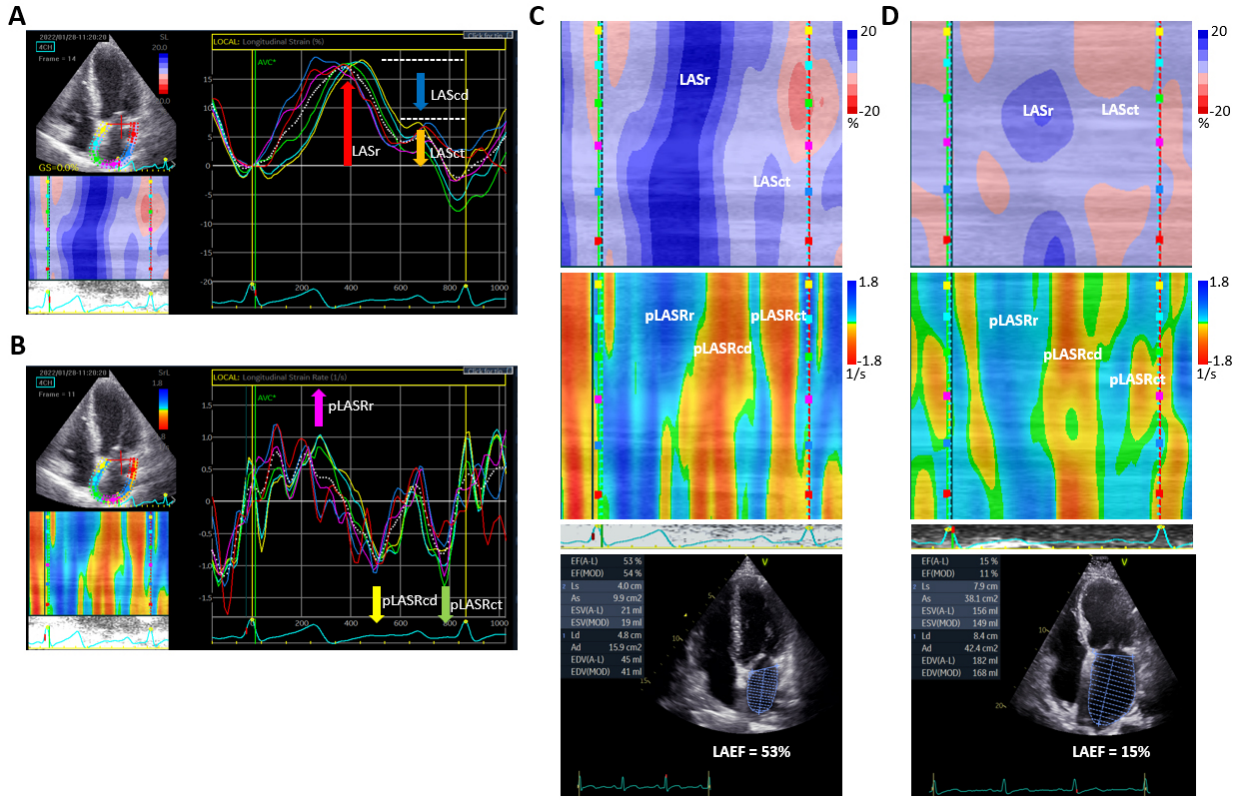
**Transthoracic echocardiography in the apical four-chamber view showing left atrial (LA) longitudinal strain and strain rate**. (A) LA strain in a paroxysmal atrial fibrillation (PAF) patient 1-day post ablation. Taking the R wave as the starting point, the first positive peak corresponds to the LA reservoir phase (LASr) (red arrow), the second peak corresponds to the LA contractile phase (LASct) (orange arrow), and the difference between the two peaks corresponds to the conduit phase (LAScd) (blue arrow). The traces are time–displacement displays, with the x-axis representing time and the y-axis showing myocardial shortening as negative and lengthening as positive (%). The depicted LA wall is divided into six segments marked by different colors. (B) The LA longitudinal strain rate in the same patient as in panel (A). The traces are time-velocity displays, with the x-axis representing time and the y-axis representing velocity (s^–1^). The LA strain rate curve is composed of a positive peak at the left ventricular systole (pLASRr) (pink arrow), followed by two negative peaks: one in the early diastole phase (pLASRcd), corresponding to passive early LV filling (yellow arrow), and one in the late diastole phase (pLASRct), corresponding to atrial booster pump function (green arrow). (C) and (D) The curved M-mode color images of LA strain (upper) and strain rate (middle), and LA emptying fraction (LAEF) (bottom) in patients with PAF and non-PAF 1-day post ablation, respectively. Blue indicates positive values and red indicates negative values. Images in panel (C) show deeper blue in the strain and strain rate images during the reservoir phase, deeper red in the strain rate images, and more homogeneous patterns of color distribution than those in panel (D), indicating better LA mechanical deformation and synchrony in PAF than non-PAF. In addition, LAEF is larger in panel (C) than that in panel (D) (53% vs. 15%), implying a good correlation between LA deformation and LAEF.

Speckle-tracking echocardiography (STE) is a novel, non-Doppler echocardiographic method to measure the magnitude and rate of atrial myocardial deformation by calculating the longitudinal strain and strain rate independent of cardiac rotational motion and the tethering effect [90, 91]. Strain is a dimensionless index that reflects total deformation of the myocardium relative to its initial length during the cardiac cycle [92], expressed as a positive value for lengthening or a negative value for shortening. STE tracks the natural acoustic markers within a region of interest (kernel) frame-by-frame, evaluating the geometric shift of each kernel throughout the cardiac cycle [36]. Fig. 1A shows an example of LA strain via the apical 4-chamber view. LA strain reaches its maximal value just before the mitral valve opening, and LA strain during the reservoir phase (LASr) is measured as the strain value at the mitral valve opening minus that at the ventricular end-diastole (a positive wave occurring during the ventricular systole) [90]. When the LA conduit phase begins, LA volume gradually decreases to a plateau until the 2nd late peak, just before the onset of the active atrial contractile phase. The strain value at the onset of atrial contraction minus that during the mitral valve opening is a surrogate of LA strain at the conduit phase (LAScd). The strain value at the ventricular end diastole minus that during the onset of atrial contraction is a surrogate of LA strain at the contraction phase (LASct). Strain rate is the rate by which the deformation occurs. Fig. 1B shows an example of LA strain rate. There is one positive peak during the reservoir phase (pLASRr) and two consecutive negative peaks during the LV diastolic phase. The first peak represents passive myocardium shortening (pLASRcd) and the second peak is the minimal value after the LA active pump phase (pLASRct). The assessment of LA strain and strain rate can use a 4-chamber view or both 4- and 2-chamber views to report the average values from 6 or 12 segments, respectively [90].

STE can be used to assess atrial fibrosis [12, 93] and LV diastolic dysfunction [58], and serve as a surrogate marker of LA remodeling to detect early LA dysfunction even prior to structural changes of the left atrium [94, 95]. Kuppahally *et al*. [96] described an inverse relationship between the degree of atrial fibrosis detected by delay-enhancement cardiac MRI and the LA strain and strain rate as shown by STE. Eichenlaub* et al*. [97] reported that LASr, LAScd, and LASct were correlated with LVA, as measured by intracardiac voltage mapping, in patients with persistent AF undergoing CA; among the three strain parameters, LASr was the most powerful predictor of atrial fibrosis. Laish-Farkash *et al*. [98] demonstrated good correlation between LASr and LA LVA as assessed by invasive intracardiac electroanatomic mapping. The LASr cutoff value of 19.7% had a sensitivity of 85.2% and a specificity of 73.3% in predicting the presence of LVA. Therefore, a reduced LA deformation during the reservoir phase may be an early marker of the extent of LA fibrosis [99], which is associated with the incidence of AF [100]. Park* et al*. [101] demonstrated that LASr was a significant predictor of new-onset AF in heart failure patients (397 of 4312 patients) regardless of the LA size.

Most cutoff values of LA strain are based on studies involving a small number of subjects and depend on age, sex, ultrasound manufacturer, and post-processing software package [102]. To establish age- and sex-based normative values of LA strain in the general population and to assess the prognostic yield of lower limits of normal LA strain in relation to future AF, a substudy of the fifth Copenhagen City Heart Study evaluated 1641 healthy participants and reported the median values (and the corresponding limits of normality) for LASr, LAScd, and LASct were 39.4% (23.0–67.6%), 23.7% (8.8–44.8%), and 15.5% (6.4–28.0%), respectively [103]. These values were similar to the results of the meta-analysis by Pathan *et al*. [104], which showed cut-off values of 39% for LASr, 23% for LAScd, and 17% for LASct in healthy adults. To investigate whether LA strain can be used to predict new-onset AF in the general population, Hauser *et al*. [105] conducted a prospective longitudinal study including 3590 participants from the fifth Copenhagen City Heart Study. Compared to the reference group (patients with LASr ≥23%), the HRs of new-onset AF were 4.16, 6.58, and 22.14 for the subgroups of patients with LASr between 23% and 19%, 19% and 15%, and <15%, respectively. Moreover, for the 2701 participants with normal LA size and preserved LV ejection fraction and without previous ischemic heart disease, LASr (HR 1.06, 95% CI 1.03–1.09) and LASct (HR 1.08, 95% CI 1.04–1.12) remained independent predictors of AF development in multivariable Cox regression analysis. Similarly, Petre *et al*. [106] revealed that LASr ≤19% and LASct ≤8.7% identify patients with new-onset AF in a population with hypertension.

In addition to enabling the identification of patients with a history of AF, STE provides prognostic information for the risk stratification of AF patients undergoing CA. Hammerstingl *et al*. [107] demonstrated that LASr was significantly reduced in patients with recurrent AF compared to those without AF recurrence. Motoki *et al*. [108] demonstrated that a low LASr at a cutoff value of 23.2% could predict the status of sinus rhythm maintenance after CA with a sensitivity of 76% and a specificity of 66%. Parwani* et al*. [109] demonstrated that a LASr cutoff value of 10% predicted post-CA AF recurrence with a sensitivity of 97.9% (95% CI 88.9–99.6%) and a specificity of 78.2% (95% CI 65.6–87.1%). One meta-analysis study including 12 studies and a total 1025 AF patients revealed that LASr was a significant predictor of post-CA AF recurrence by multivariable pooled analysis (OR 1.16, 95% CI 1.09–1.24) [110]. In addition to LASr, LASct is also reported to be associated with the outcome of AF ablation. Wen *et al*. [111] demonstrated that LASct is an independent risk factor for AF recurrence; the 5-year cumulative recurrence probability was much higher in patients with LASct ≥–12% than in those with LASct <–12% (87.6% vs. 52.9%, log rank *p *< 0.0001). Eichenlaub *et al*. [97] reported that LASr and LASct were both predictors of AF recurrence after CA in patients with persistent AF. Thus, LA deformation abnormalities consistently predict recurrence of AF after CA although the cutoff values of deformational parameters vary among studies.

Even if those with LAD >50 mm have a four-fold higher risk of developing AF [29], some patients with severe LA dilatation do not have AF. A recent systemic review and meta-analysis by Bajraktari *et al*. [26] revealed that the strongest LA predictor of AF recurrence after CA was LASr <20%, followed by LAD ≥50 mm and ${\mathrm{LAV}}_{\text{max}}$ >150 mL. This result suggests that LA dysfunction plays a more pivotal role than LA enlargement in the development of AF. Recently, our group demonstrated that LAEF, ${\mathrm{LAV}}_{\text{min}}$, LASr, pLASRr, and pLASRct were associated with the occurrence of AF, and multivariate regression analysis revealed that pLASRct was the only independent factor associated with the absence of AF in those with LAD ≥50 mm [112]. Atrial booster pump function represents the inherent contractility of the LA myocardium. Previous studies have revealed that LV diastolic dysfunction is associated with impaired LA reservoir and conduit functions in the presence of an increased LA contractile function [113, 114]. When LA reservoir function is impaired, LA booster pump function would be enhanced to compensate for the reduced LA emptying volume. Thus, a reduced pLASRct indicates a more advanced stage of diseased atrial myocardium because pLASRr and pLASRcd have been reduced at a earlier stage. Furthermore, because LA reservoir and conduit functions represent intrinsic LA relaxation and are partly affected by LV systolic performance, LA booster pump function may be the most sensitive predictor of AF occurrence [115] and is effective in predicting AF genesis and recurrence [41, 116].

Even if STE provides a feasible and reproducible assessment of LA function, STE is dependent on the quality of echocardiographic images and frame rates, and requires time-consuming offline analysis. Therefore, it may not suitable for all clinical settings [117]. In addition, intervendor discordance of LA strain assessed by STE remains a problem to be solved. For example, LA reservoir strains differ significantly by using different speckling tracking analysis systems (GE vs. Siemens) [108]. The intervendor/intersoftware variability should be considered when discussing published LA strain values.

### 2.7 LA Mechanical Dispersion

LA electrical and mechanical dysfunction coexist in the early phase before LA enlargement [118]. Sarvari *et al*. [119] demonstrated that inhomogeneous contraction of the left atrium potentially predicted AF recurrence after ablation. Because STE is angle-independent and can assess regional myocardial function and timing accurately, the regional differences in 2-dimensional (2D) STE-derived LA strain and strain rate potentially could be used to measure heterogeneous LA fibrosis and dysfunction indirectly. LA mechanical dispersion is calculated as the standard deviation in time to peak strain of the LA segments [119]. It is greater in AF patients than in healthy individuals, increases proportionately to the duration of AF [116], and provides prognostic information on the risk of AF recurrence in patients after ablation [116, 119]. In a case-control study, patients with new-onset AF had significantly worse LASr and LASct, and more pronounced LA mechanical dispersion, than those without AF [120]. However, it is time-consuming to calculate the standard deviation for parameters of LA mechanical dispersion because sophisticated mathematics is needed for averaging the 2–3 instances of six segmental values per apical 4-chamber and 2-chamber views (3 peaks of LA strain rate curve in sinus rhythm). Alternatively, the curved M-mode color images of LA strain and strain rate provide detailed spatial and temporal information on LA deformation mechanics. These images provide a unidimensional view of LA strain and strain rate, illustrating the changes in length and in strain/sec of the depicted LA wall along the time axis, respectively. As shown in Fig. 1C–D, the spatial and temporal information of LA deformation can be displayed in these images, on which blue or red color, deep or light hue, and pattern of color distribution indicate the direction, strength, and homogeneity of LA deformation, respectively. However, it is challenging to use visual estimation to precisely differentiate these images. Recently our group demonstrated that a deep convolutional neural network (CNN) analysis can successfully incorporate spatial and temporal features from these STE images into an overall assessment of LA deformation mechanics; indeed, the STE image-based CNN model outperformed the logistic regression model using LAD, LAEF, LA strain, and strain rate in predicting AF recurrence after CA [121]. This study demonstrated the potential advantages of supervised deep learning with CNNs to classify images to provide prognostic information for AF intervention. Note that this retrospective study included only 606 patients, and large prospective studies are needed to optimize CNN model performance. Recently, manufacturers have begun developing dedicated software packages for LA strain measurement after publication of the common standards to assess LA strain [90]. Newly-available softwares, such as AutoStrain (TomTec) or LA Automated Function Imaging (Echo-Pac), allow for a quick assessment of LA strain. Future goals would be to achieve fully automatic generation and interpretation of LA STE images, provide fast and reproducible assessment of LA deformation properties, and validate and enhance the performance of CNN models in this domain.

### 2.8 Reverse Remodeling after Cardiac Ablation for AF

LA substrate modification in addition to pulmonary vein isolation improves AF ablation outcome [122, 123, 124]. Maintenance of sinus rhythm leads to histological reverse remodeling and functional recovery, shown by reduced LA size, improved LA function, and increased LA conduction velocity [55, 125]. However, LA ablation itself impairs LA function, a result related to the extent of scarring [126]. As a result of the different degrees of myocardial damage associated with the different ablation strategies and AF populations, a discrepancy exists in the literature regarding LA functional reverse remodeling after successful AF ablation. Tops *et al*. [127] found that LA structural reverse remodeling was associated with a concomitant improvement in LA strain. Spethmann *et al*. [128] demonstrated that LASr and LASct normalized within 6 months after CA in PAF patients with no AF recurrence. Perea* et al*. [129] used cardiac MRI to reveal that extensive LA linear lesions reduced LA volume and preserved or even increased LAEF in most patients after successful CA. However, Lemola* et al*. [130] found that LA linear ablation restored sinus rhythm but compromised LA systolic function in patients with PAF. A meta-analysis by Jeevanantham *et al*. [131] revealed that successful CA significantly decreased LAD and LAV without significant influences on LAEF. To evaluate the influence of CA outcome on LA reverse remodeling in the same patients, Yang* et al*. [132] studied 38 patients undergoing a repeat CA for AF recurrence after a 1st circumferential pulmonary vein isolation. The absence of LA size reduction after a 1st unsuccessful CA and the presence of significant LA size reduction after a successful second CA in the same patients imply that procedural success was associated with LA structural reverse remodeling. However, LAEF, LA strain, and LA strain rate were not concomitantly improved. Another meta-analysis by Xiong *et al*. [133] (25 studies, 2040 patients) revealed that LAEF is significantly decreased in PAF but insignificantly changed in persistent AF after CA. It is likely that differences in the extent of scarring associated with different ablation strategies, preexisting LA fibrosis, and clinical outcome contribute to variable changes in LAEF after CA between PAF and persistent AF patients. Recently, we noted significant LA reverse remodeling, evidenced by reduced LA size and improved LAEF, in non-PAF patients undergoing a successful LVA-guided LA linear ablation [123]. LA functional reverse remodeling was noted even in patients undergoing extensive LA linear ablation. Possibly, the LA linear ablation strategy targeting LVA to avoid damage to otherwise healthy LA myocardium could help preserve the effect of LA functional reverse remodeling.

Although the results are variable regarding LA functional change after successful AF ablation, LA structural reverse remodeling has been consistently observed after successful AF ablation and might be considered as a marker of freedom from AF recurrence. By using different variables and definitions, Kagawa *et al*. [134] demonstrated that a reduction of ≥5% in LAD at 6 months post CA was associated with freedom from late AF recurrence in patients with persistent AF (AUC 0.653, *p *< 0.05); Maille *et al*. [130] demonstrated that patients with a ≥15% reduction in ${\mathrm{LAV}}_{\text{max}}$ after CA had markedly less AF recurrence; Kawakami* et al*. [135] demonstrated that LAV normalization (defined as LAVI of ≤34 mL/$m^{2}$) at follow-up was significantly associated with a better long-term outcome of AF ablation compared to patients who did not meet this standard. It seems necessary to clearly define LA structural reverse remodeling in order to evaluate the impact of LA reverse remodeling on the long-term outcome of AF ablation.

Compared with cross-sectional observational studies, longitudinal studies can avoid time-invariant unobserved individual differences, detect changes in parameters beyond a single moment in time, and establish sequences of events to suggest cause-and-effect relationships. Our group conducted a three-year longitudinal study to evaluate the long-term prognostic influence of LAD remodeling on the outcome of AF ablation. We found that a longitudinal linear mixed model-based two stage model outperformed a logistic model using the baseline LAD in classifying outcome status after AF ablation [136]. In addition, LAD was shortened over the first 3 months and remained stable up to 36 months after CA. Similarly, Reant *et al*. [137] also found a reduction in LAD during the first 3 months after CA, which then remained stable up to 12 months post CA. The degree of LAD reduction was significantly influenced by the baseline LAD [136]. Interestingly, LAD was reduced in both the success and failure groups. Because the ablation lesions themselves also decrease LA size [138], the prognostic value of LAD reduction in predicting the outcomes of AF ablation remains a matter that needs clarification. In addition, further longitudinal studies of LA functional remodeling may unveil the long-term prognostic influence of the extent of reversibility of LA deformation parameters on AF ablation outcome.

### 2.9 Three-Dimensional Echocardiography

Three-dimensional (3D) echocardiography is a novel approach providing a non-invasive method to analyze cardiac anatomy and function. The measurement of LAV by 2D echocardiography is based on geometric assumptions, which often results in underestimation of LAV compared with that measured using cardiac MRI. 3D echocardiography provides a more accurate measure of LAV because of automated border detection, the acquisition of 3D data sets at different phases of the cardiac cycle, and more accurate assessment of asymmetric remodeling of the left atrium [139, 140, 141]. Badano *et al*. [142] revealed that LAD and area measurements significantly underestimated actual LA size and misclassified the grade of severity of LA dilatation in 43–70% of patients if 3D LAV was used as the gold standard. In addition, 3D echocardiography provides unique measurement of phasic changes of LAV during the cardiac cycle and detailed information of the different LA functions [143]. Marsan *et al*. [144] demonstrated that a significant reduction of ${\mathrm{LAV}}_{\text{max}}$ and improvement in LA active contraction and reservoir function were noted in the success CA group but not in the AF recurrence group three months after CA. Schaff *et al*. [145] revealed that LAVI and LA function assessed by 3D echocardiography had higher discriminating power than 2D echocardiography in identifying PAF.

LA myocardial fibers are arranged not only in the longitudinal direction, and LA fibrosis may occur heterogeneously in patients with AF [98]. Studies have shown that 3D-STE-derived circumferential, longitudinal, radial, as well as area strain are significantly reduced in patients with AF compared to matched controls [146, 147]. Because 2D-STE only provides longitudinal deformation information, some LA dysfunctions may be overlooked by 2D-STE.

3D-STE has the advantage of combining longitudinal and circumferential strain information [147], and a few studies have demonstrated the superiority of 3D-STE over 2D-STE in predicting AF occurrence or recurrence after CA [145, 147, 148]. Moreover, 3D-STE can be used to detect LA functional reverse remodeling by showing improvement of global strain and LA dyssynchrony [149]. Theoretically, 3D-STE also has the advantage of overcoming the out-of-plane motion that may occur with 2D-STE, as the advent of 3D acquisition allows tracking of speckles in the myocardium in the 3D space [150]. However, 3D echocardiography is limited by the slow temporal resolution and motion artifacts, and evaluation of the clinical utility of 3D-STE remains insufficient. Further studies are needed to clarify whether the diagnostic and prognostic value of 3D-STE is superior to that of 2D-STE [151].

## 3. Conclusions

Echocardiography is a safe and non-invasive technique providing quantitative analyses of cardiac chamber size and function, but clinical measurement of the left atrium has so far been limited to evaluation of LAD and LAV. Considerable data support the use of LAEF to predict incident AF and AF recurrence after CA. STE enables early detection of LA dysfunction before anatomical changes and also helps identify patients with a severely dilated left atrium at risk for AF. The studies discussed in this review support the contention that LAEF and LA strain provide optimal diagnostic and prognostic information for assessing AF patients. It is likely that future guidelines for patient evaluation and guidance of AF ablation will include evaluation of not only LA chamber size but also LA function parameters. Compared with cardiac MRI, echocardiography provides a real-time and feasible method to assess LA function (${\mathrm{LAVI}}_{\text{min}}$, LAEF and LA strain). Improvements in temporal and spatial resolution, automation and standardization among platforms and vendors will enhance the utility of LA strain indices in the near future. Histological and functional reverse remodeling after resuming sinus rhythm may bring anatomical and functional recovery of the left atrium. However, discrepancies regarding LA functional reverse remodeling after successful AF ablation persist, and a clear definition of LA structural reverse remodeling is still lacking. A longitudinal study of the long-term prognostic impact of LAD remodeling on the outcome of AF ablation revealed that LAD was reduced regardless of the outcome of AF ablation, and the degree of LAD reduction was significantly affected by the baseline LAD. Definitely, robust clinical outcomes data from large perspective trials using longitudinal studies are needed to understand the natural history of LA structural and functional reverse remodeling as well as the impact of such changes on the outcome of AF ablation. LA mechanical dispersion provides prognostic information on AF risk, and the curved M-mode color images of LA strain and strain rate provide detailed spatial and temporal information on LA deformation mechanics. Deep CNNs overcome subjective visual assessment to aid image-based outcome classification. Therefore, it is promising that the development of fully automated generation and interpretation of LA STE images with well-trained deep learning classifiers will provide more rapid and reproducible assessment of LA deformation properties. 3D echocardiography provides valuable information on LA size, phasic functions and myocardial mechanics. New developments in hardware technology will overcome the limitations of lower spatial and temporal resolution of 3D echocardiography.

## Abbreviations

2D, 2-dimensional; 3D, 3-dimensional; AF, atrial fibrillation; AUC, area under the curve; CA, catheter ablation; CI, confidence interval; CNN, convolutional neural network; HFpEF, Heart failure with preserved ejection fraction; HFrEF, Heart failure with reduced ejection fraction; HR, hazard ratio; LA, left atrial; LAD, left atrial dimension; LAEF, left atrial emptying fraction; LAV, left atrial volume; LAVI, LAV index; LAScd, conduit left atrial strain; LASct, contractile left atrial strain; LASr, reservoir left atrial strain; LV, left ventricular; LVA, low voltage area; OR, odds ratio; PAF, paroxysmal atrial fibrillation; pLASRcd, peak conduit left atrial strain rate; pLASRct, peak contractile left atrial strain rate; pLASRr, peak reservoir left atrial strain rate; STE, speckle-tracking echocardiography; TACT, total atrial conduction time.
